# Contamination analysis of Arctic ice samples as planetary field analogs and implications for future life-detection missions to Europa and Enceladus

**DOI:** 10.1038/s41598-022-16370-5

**Published:** 2022-07-27

**Authors:** Lígia F. Coelho, Marie-Amélie Blais, Alex Matveev, Tina Keller-Costa, Warwick F. Vincent, Rodrigo Costa, Zita Martins, João Canário

**Affiliations:** 1 Department of Chemical Engineering, Centro de Química Estrutural, Institute of Molecular Sciences, Instituto Superior Técnico, Universidade de Lisboa, Av. Rovisco Pais 1, 1049-001 Lisbon, Portugal; 2 Institute for Bioengineering and Biosciences, Instituto Superior Técnico, Universidade de Lisboa, Av. Rovisco Pais 1, 1049-001 Lisbon, Portugal; 3 Associate Laboratory i4HB—Institute for Health and Bioeconomy at Instituto Superior Técnico, Universidade de Lisboa, Av. Rovisco Pais, 1049-001 Lisbon, Portugal; 4 Centre for Northern Studies (CEN), Université Laval, Québec City, QC G1V 0A6 Canada; 5 Takuvik Joint International Laboratory, Département de Biologie, Université Laval, Québec City, QC G1V 0A6 Canada; 6 Department of Geography and Environment, Concordia University, Montréal, QC H4B 1R6 Canada; 7 Centro de Ciências do Mar (CCMAR), Universidade do Algarve, 8005-139 Faro, Portugal

**Keywords:** Astrobiology, Rings and moons

## Abstract

Missions to detect extraterrestrial life are being designed to visit Europa and Enceladus in the next decades. The contact between the mission payload and the habitable subsurface of these satellites involves significant risk of forward contamination. The standardization of protocols to decontaminate ice cores from planetary field analogs of icy moons, and monitor the contamination in downstream analysis, has a direct application for developing clean approaches crucial to life detection missions in these satellites. Here we developed a comprehensive protocol that can be used to monitor and minimize the contamination of Arctic ice cores in processing and downstream analysis. We physically removed the exterior layers of ice cores to minimize bioburden from sampling. To monitor contamination, we constructed artificial controls and applied culture-dependent and culture-independent techniques such as 16S rRNA amplicon sequencing. We identified 13 bacterial contaminants, including a radioresistant species. This protocol decreases the contamination risk, provides quantitative and qualitative information about contamination agents, and allows validation of the results obtained. This study highlights the importance of decreasing and evaluating prokaryotic contamination in the processing of polar ice cores, including in their use as analogs of Europa and Enceladus.

## Introduction

Europa and Enceladus are two icy moons from our solar system identified as ocean worlds due to the presence of a liquid ocean under their icy surface^1,2^. Europa has also secondary liquid water reservoirs, perched in the ice and closer to the active surface^3^. The water bodies present in these satellites are considered habitable environments^4^. Several concepts for future lander missions to these moons have been developed; e.g., Europa Lander, Enceladus Orbilander, and Joint Europa Mission (JEM)^5–7^. These missions will need to drill and sample a layer of ice (exact extension still undetermined) to eventually reach interface water.

The Committee of Space Research (COSPAR) recommends that the study of methods of bioburden reduction for these missions should reflect the type of environments found on Europa or Enceladus, focusing on Earth organisms most likely to survive on these moons, such as cold and radiation tolerant organisms^8–10^. Environments on Earth that exhibit similar extreme conditions as planets and moons in our solar system are called planetary field analogues^11–13^. Both the Arctic and Antarctic offer locations that mimic environments present in the icy moons of Jupiter and Saturn^12^. These locations are populated by extremophiles—organisms adapted to survive these severe conditions such as extreme temperature and pH, dryness, oxidation, UV radiation, high pressure, and high salinity^14,15^. Microbes from these habitats are viable despite hundreds to thousands of years in terrestrial glaciers and cryo-permafrost environments^16,17^, increasing the plausibility of finding putative life forms in Europa and Enceladus. Polar extremophiles are also known to survive sterilization procedures for planetary protection^18^ and under space conditions on-board the International Space Station (ISS)^19^.

The challenges of sampling, processing, and analyzing Arctic and Antarctic ice samples are the closest to those of future life-detection missions in these satellites^20,21^. The constraints include difficulties in sampling, analysis of low biomass samples, and the need to minimize and monitor potential sources^22^ of contamination from mesophilic environments on Earth, where microbes are ubiquitous and in high abundance.

When studying recent terrestrial ice, contamination is critical, and sources are mainly due to equipment such as ice corers, handling, and transportation^20,23,24^. In the context of planetary field analogs, we add more contamination sources such as snow, air, and soil microbes that are part of the atmospheric and soil microbiome but are not expected to be present in icy worlds with thin atmospheres and no regolith^25^. Studies from the last 20 years of ice core research mention the use of sterile equipment in the field while drilling the ice cores (Table S1—Supplementary material). Codes of conduct and clean protocols to sample pristine subglacial lakes in Antarctica have also been created recently^22,24^, which represents a challenging and laudable effort by the scientific community to conserve these unique microbial ecosystems. However, the potential sources of contamination are not limited to the field. During manipulations of ice cores in the laboratory, microbial contaminants can be introduced from the laboratory air, equipment, materials, reagents, or even by humans during downstream analysis such as filtration, DNA extraction (the kitome), amplification, and sequencing, or during cultivation in a nutritive medium. These procedures will be robotized in lander space missions; however, they still represent more layers of contact between man-made equipment coming from Earth and extraterrestrial samples. Sterilization methods used for equipment cannot be directly applied on ice samples^21^. Controlled heat, UV-C, and chemical disinfectants such as ethanol, benzethonium chloride, and sodium hypochlorite have been used directly in the exterior of ice samples being especially efficient in reducing active contaminants in cultivation work^20,23,26^. However, they are not adequate for life detection, molecular methods of low-biomass ice samples, or the integrity of other microbial analyses. For example, ethanol is an effective disinfectant to decrease contamination for culture-dependent analysis, however, it does not destroy DNA molecules^20^. While excising the external layers of the ice cores has proved effective in removing most contaminating cells^20,23,26,27^, without compromising the interior native biota, no known protocol can completely prevent contamination, which leads us to the last possible resource for an ethical sampling and processing methodology: contamination monitoring through the use of background controls^27^ that have proved to be very effective^27,28^. Culture-dependent and -independent analyses^9,27,29–39^ have been used in background controls and in the planetary protection context. In past studies on ice cores, culture-dependent investigations appeared to instigate more care to prevent contaminants in comparison with culture-independent techniques (Table S1—Supplementary material). This is likely because DNA contamination from the laboratory air or sterile material is commonly considered insignificant, due to its presumably low representation in comparison with the microbial load of the whole community present in the samples, which is now overcome by the increased sensitivity of polymerase chain reaction (PCR) and DNA sequencing techniques. The lack of standardized methodology adopted to decrease and monitor contaminations in ice core analysis, similar to what already exists to sampling^22,24^, remains a limiting issue for the scientific integrity of the acquired data in icy planetary field analogs, as well as for the design of proven and robust protocols for the future lander and return missions to icy moons. As a result, the identity and function of microbial contaminants expected from ice core analysis remains a challenge in the field of planetary protection.

**Figure 1:**
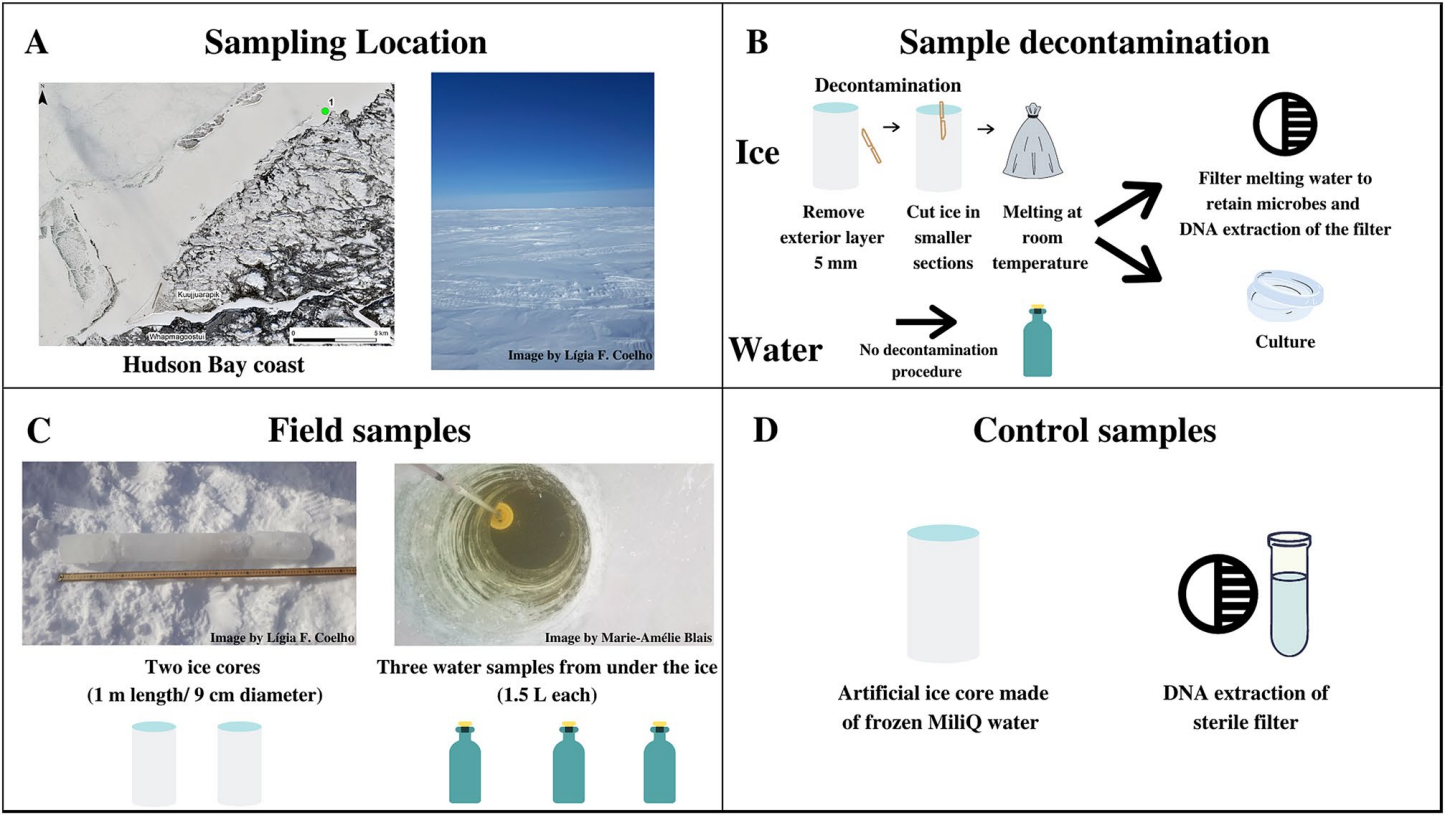
(**A**) Sampling location on the east coast of Hudson Bay, Quebec, Canada, latitude 55.39° N; longitude 77.61° W (Map data © Sentinel-2), and the salinity measured on-site (% by mass). (**B**) Sampling decontamination procedure and following processing preceding culture-dependent and culture-independent analysis. (**C**) Description of environmental samples with respective replicates: ice cores (duplicate—Ice 1, Ice 2), and interface water (triplicate—Water 1, Water 2, Water 3); (**D**) Control samples: an artificial sterile ice core made in the laboratory referred as a processing control, and a clean filter inside a clean microtube used as a control for downstream DNA analysis referred as DNA extraction control.

In this study, we propose a multidimensional approach to restrict and monitor the contamination inherent in the processing and analyzing ice samples, combining the most effective methods presented in the last 20 years on ice core studies (Table S1—Supplementary material). The decontamination methodology for the ice core surface was mechanical to decrease contaminants while preserving the natural biota. We constructed background controls (Fig. 1): an artificial ice core made of sterile MilliQ water (processing control) and a DNA extraction control sample (DNA-extraction control). We used both culture-dependent and -independent techniques, such as 16S rRNA gene amplicon sequencing of metagenomic DNA samples, accessing several levels of visible and quantifiable prokaryotic contamination. We identified the contaminating microorganisms of the present study using an established ribosomal marker database to understand the astrobiology relevance of the contaminants. Such a decontamination protocol would be suitable for the design of life-detection experiments on planetary field analogs of Europa and Enceladus, targeting ice cores that may serve as a proxy for habitats of putative extraterrestrial communities. Also, our protocol will serve as a testbed for procedures of decontamination of samples from future landing/return missions to the icy moons.

## Results and discussion

### Assessment of ice core contamination using cultivation-dependent and independent techniques

No colony forming units (CFUs) were registered on the processing control plates, during the 30 days of the experiment (Table 1-A). The interface water samples had notable more culturable bacteria than the sea ice samples, an expected result since usually winter sea ice has relatively low biomass^28^. This suggests that the culture methodologies adopted were appropriate for the environmental samples used, validating the results of the processing control.

**Table 1:** Cultures results (**A**) and flow cytometry results (**B**) are presented in average colony-forming units (CFUs) and cells per mL, respectively, of water (interface water), melted environmental ice cores (ice meltwater), and processing control. Environmental water culture results (**A**) and flow cytometry results (**B**) were partially adapted from Coelho et al. (2022)^28^ on the same ice cores. **(C)** DNA quantification (ng/µL) results on environmental water and ice and the processing control based on fluorescence (Qubit fluorometer).

**(A) Cultures (CFU mL**^**−1**^**)**						
Days after culturing	Number of CFU/mL of water (× 10^2^)	Number of CFU/mL of ice meltwater (× 10^2^)	Processing control			
5	400 ± 1	0.6 ± 0.02	0			
10	30 ± 1	3 ± 0.1	0			
15	50 ± 1	3 ± 0.7	0			
20	20 ± 1	2 ± 0.4	0			
25	30 ± 1	2 ± 0.6	0			
30	10 ± 3	0	0			
Total	500 ± 20	10 ± 2	0			

**(B) Flow cytometry (cells mL**^**−1**^**)**						
Number of cells/mL of water (× 10^4^)	Number of cells/mL of ice meltwater (× 10^4^)	Processing control (× 10^4^)				
36 ± 4	3.4 ± 1.0	0.3				

**(C) DNA quantification (ng µL**^**−1**^**)**						
Ice 1	Ice 2	Water 1	Water 2	Water 3	Processing control	DNA-extraction control
0.9 ± 0.1	0.4 ± 0.0	0.9 ± 0.1	1.1 ± 0.0	0.1 ± 0.1	DL	DL

*CFU* colony-forming unit, *DL* Below detection limit.

The culture-independent cell counting method reported the presence of what could be prokaryotic cells in the processing control (Table 1-B). The whole prokaryotic cell counts quantified in the control was only 0.8% of the total prokaryotic cell count quantified in interface water, and 8.8% of prokaryotic cell count quantified in ice. This shows how decontamination following the use of appropriate background controls is more crucial when studying ice than water since the magnitude of bacterial contamination is expected to be higher for low-biomass samples.

Although not culturable, these contaminating cells may still have their DNA undamaged and thus contaminate the downstream DNA analysis (DNA extraction, amplification, and sequencing). The DNA quantification methodology used (Table 1-C) accounted for DNA in all environmental samples (however in low quantities), but not in the background controls. These results suggest that flow cytometry results of the processing control were either an “artifact” or, that DNA in this sample was present in very low concentration and thus undetectable by standard fluorometric DNA quantification techniques with a detection limit of 10 pg/µL. This is significant considering that following this protocol there will be less contaminating noise on DNA-detection fluorometric techniques—a possible tool to search for extraterrestrial life in future missions^5^.

### Amplification and sequencing of 16S rRNA gene fragments detected prokaryotic contaminants in both background controls

**Figure 2:**
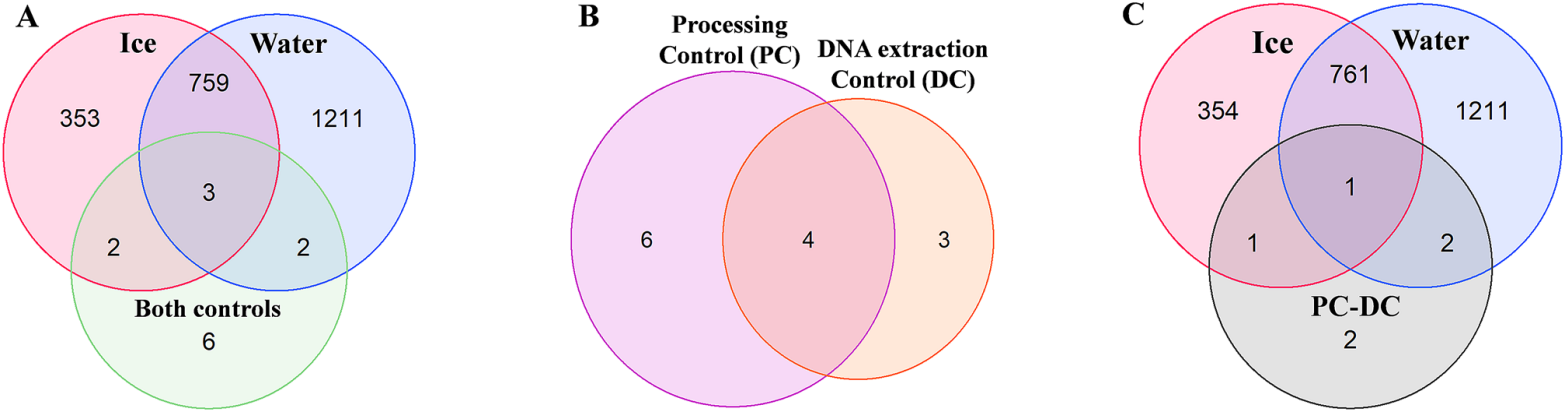
Specificity and sharedness of prokaryotic OTUs across environmental samples (interface water and ice) and controls. Venn diagrams display the number of (**A**) OTUs common and specific to environmental water, environmental ice, and both control samples, (**B**) OTUs common and specific to the processing control (PC) and the DNA extraction control (DC), and (**C**) OTUs common and specific to environmental interface water and ice samples comparing with PC-DC (contaminant OTUs exclusive from processing control). In diagrams (**A**) and (**C**) replicate samples within each sample type were pooled together.

Even though our control samples have presented undetectable DNA concentrations (Table 1-C), amplicon sequencing of the 16S rRNA gene revealed the presence of prokaryotic DNA present in both the processing control and the DNA-extraction control (Fig. 2). The DNA sequences from the two controls were clustered in 13 Operational Taxonomic Units—(OTUs) based on 97% nucleotide similarity, which works as a proxy for prokaryotic species. Given their presence in the controls, we labeled them “contaminating OTUs”. Seven of these OTUs were also present in the environmental samples (Fig. 2A), although they represented collectively only less than 0.6% of the total number OTUs identified (Table 2). From these seven contaminating OTUs, two appeared only in sea ice samples, representing collectively 0.2% (Table 2) of the total number of sea ice OTUs; two were only in water samples, representing collectively 0.1% of the total number of water OTUs (Fig. 2A); and three were present in both ice and water environmental samples.

**Table 2:** Relative abundance (%) of contaminating, prokaryotic OTUs in environmental ice (Ice 1, Ice 2), and interface water (Water 1, Water 2, Water 3).

OTU_ID	RDP classifier (> 70%)	Ice 1	Ice 2	Water 1	Water 2	Water 3	Average
OTU_4	*Stenotrophomonas*	0	0.02	0	0	0	0.004 ± 0.01
OTU_5	*Corynebacterium*	0.07	0.09	0	0	0	0.03 ± 0.03
OTU_19	*Pseudomonas*	0.09	0.04	0.004	0.009	0.02	0.03 ± 0.03
OTU_22	*Sulfurospirillum*	0	0	0	0	0	0
OTU_25	*Acinetobacter*	0.03	0	0.06	0.20	0.2	0.1 ± 0.09
OTU_36	*Asprobacter*	0	0	0	0	0.008	0.002 ± 0.00
OTU_63	*Escherichia*/*Shigella*	0	0	0	0	0	0
OTU_70	*Chitinophagaceae* family	0	0	0	0	0	0
OTU_98	*Burkholderia*	0	0	0	0	0.008	0.002 ± 0.00
OTU_103	*Sulfurimonas*	0	0	0	0	0	0
OTU_167	*Aridibacter*	0	0	0	0	0	0
OTU_209	*Acinetobacter*	0	0	0	0	0	0
OTU_1153	*Sulfurospirillum*	0.07	0.17	0.06	0.18	0.35	0.17 ± 0.10
Total contamination abundance		0.25	0.32	0.12	0.40	0.61	0.3 ± 0.2

While the processing control had ten contaminating OTUs, the DNA-extraction control had seven (Fig. 2B). The processing control was in direct contact with the same type of materials that were also used to process the environmental samples such as the ice saw, filtration system, sterile bags, and gloves, which could be the origin of its exclusive contaminating OTUs (six). The DNA-extraction control contaminants could be from the extraction reagents, the laboratory environment, the personnel, the plastics as microtubes, and pipette tips^40^, even though plastics were treated to be DNA-free by the company. Another possible source of contamination for the processing control and the DNA-extraction control is the samples. Cross-contamination could occur during filtration (for the processing control), or DNA extraction (for the processing control and DNA-extraction control)^27,40^, since both methodologies concentrate a great quantity of biological material that will increase bioburden in the laboratory surfaces and air for a determined period. However, the small absolute quantity of contaminants detected in the samples discourages such conclusions.

Both controls shared four of the 13 contaminants. To account only for processing contaminants, the OTUs of DNA-extraction control were in silico subtracted from processing control, remaining only exclusive contaminant OTUs from processing (“PC-DC”-Fig. 2C). The results for only the processing control contaminants show that interface water maintained two shared OTUs with the control while ice now only just shared one contaminant OTU. One OTU remains ubiquitous in all samples. Predictably, interface water was much richer in biomass than ice (Fig. 2A), both native and contaminant (Table 2) wherefore possible cross-contamination from interface water back to the controls, during processing, was more likely than from the sea ice.

### Taxonomic profiles of background controls

**Figure 3:**
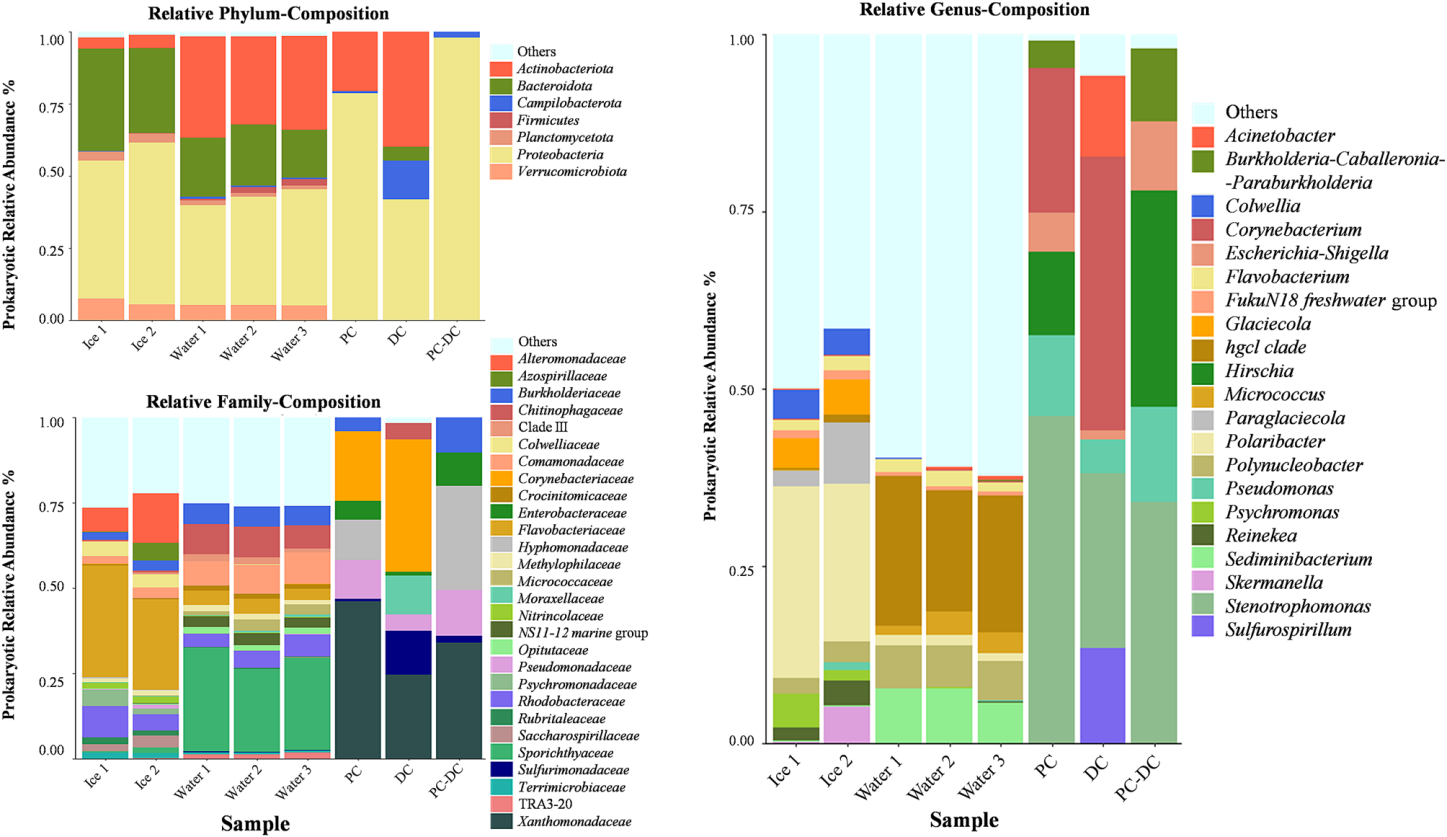
Taxonomic composition of prokaryotic communities in environmental samples (ice and interface water, including replicates) as well as controls (processing control (PC), DNA extraction control (DC), and contaminant OTUs exclusive from processing control (PC-DC)), based on the relative abundance of OTUs of the non-rarefied dataset. For improved readability, taxa below 0.5% of relative abundance were combined under the category “Others”.

While contaminants represented a small group of just a few different prokaryotic identities (OTUs) and were very rare in environmental samples (Fig. 2 and Table 2), the load of total contaminating DNA strands in controls was considerable (Fig. 3). These results agree with the normal results of amplicon sequencing analysis to monitor reagent contamination which has been described to have taxon richness inversely correlating with the bacterial load^41^. Meaning that the contaminant community profile is usually distinguishable from the rich, and diverse environmental community, which we also see in our dataset (Fig. 3). The in-silico decontamination (exclusion of contaminants from environmental analysis outputs^40^) (Fig. S1—Supplementary Material) shows how evident some contaminants are (e.g., *Acinetobacter*) versus others less abundant which were grouped in “Others”. Similar results were reported in past studies on decontamination methods for low biomass samples^27^. The parallels between the taxonomic profiles of the environmental samples and controls stop at lower taxonomic ranks (order and genus)—Fig. 3. Some contaminating OTUs of the genera *Acinetobacter* and *Corynebacterium* were present, although not abundant, in the environmental samples (Ice 1, Ice 2, Water 2, and Water 3)—Fig. 3. In the controls, *Acinetobacter* was more abundant in the DNA-extraction control. Members from the *Acinetobacter* genus have been identified before as part of the kitome (i.e., contaminants from DNA extraction reagents)^41^, and as contaminants of the NASA Space Assembly Facility^42,43^, where the last cleaning steps of space vehicles take place before launch. Finally, the *Acinetobacter* case shows how our method allows not only the reduction, identification, and monitoring of likely contaminants but also a close trace to their possible origin.

Silva taxonomy (https://www.arb-silva.de/aligner/), as well as RDP database assignments, showed that OTUs were from the genera *Burkholderia*-*Caballeronia*-*Paraburkholderia*, *Acinetobacter*, *Escherichia-Shigella*, *Corynebacterium*, *Hirschia*, *Pseudomonas*, *Stenotrophomonas,* and *Sulfurospirillum* (Tables 2 and 3). Previous studies reported *Corynebacterium*, *Burkholderia*, *Acinetobacter,* and *Pseudomonas* to be present in other background controls constructed from artificial sterile ice cores and DNA extraction blanks^27,41^, and another study report *Stenotrophomonas* and *Acinetobacter* to be able to grow after bioburden assays performed by ESA which include heat-shock treatment^9^.

**Table 3:** List of all OTUs retrieved in the processing control (PC) and DNA extraction control (DC). The OTU’s abundance (number of reads) are presented from environmental ice meltwater (Ice 1, Ice 2), environmental interface water (Water 1, Water 2, Water 3), PC, DC, and PC-DC (removing PC shared OTUs with DNA-extraction control remaining only contaminant OTUs exclusive from the processing control).

OTU_ID	RDP classifier (> 70%)	Closest RDP* type strain (Accession number)	a_b probability score	Number of reads in Ice 1	Number of reads in Ice 2	Number of reads in Water 1	Number of reads in Water 2	Number of reads in Water 3	Number of reads in Processing control (PC)	Number of reads in DNA amplification control (DC)	PC-DC
OTU_4	*Stenotrophomonas*	*Stenotrophomonas maltophilia* (T); ATCC 19,867; AB021405	0.918	0	2	0	0	0	8506	6088	2418
OTU_5	*Corynebacterium*	*Corynebacterium tuberculostearicum* (T); CIP107291; AJ438050	0.966	5	10	0	0	0	3770	9522	0
OTU_19	*Pseudomonas*	*Pseudomonas proteolytica* (T); type strain: CMS 64; AJ537603	0.954	7	4	1	2	4	2122	1174	948
OTU_22	*Sulfurospirillum*	*Sulfurospirillum alkalitolerans* (T); HTRB-L1; GQ863490	0.742	0	0	0	0	0	0	3169	0
OTU_25	*Acinetobacter*	*Acinetobacter guillouiae* (T); DSM 590; X81659	0.962	2	0	14	46	57	0	2771	0
OTU_36	*Asprobacter*	*Asprobacter aquaticus* (T); DRW22-8; KF056993	0.947	0	0	0	0	2	2165	0	2165
OTU_63	*Escherichia/Shigella*	*Shigella sonnei* (T); type strain: CECT 4887; FR870445	0.977	0	0	0	0	0	1004	310	694
OTU_70	*Chitinophagaceae* family	*Ferruginibacter alkalilentus* (T); HU1-GD23; FJ177530	0.811	0	0	0	0	0	0	1170	0
OTU_98	*Burkholderia*	*Burkholderia stabilis* (T); LMG 14,294; AF097533	0.954	0	0	0	0	2	728	0	728
OTU_103	*Sulfurimonas*	*Sulfurimonas denitrificans* (T); DSM 1251; CP000153	0.865	0	0	0	0	0	141	0	141
OTU_167	*Aridibacter*	*Aridibacter famidurans* (T); A22_HD_4H; KF245634	0.918	0	0	0	0	0	0	269	0
OTU_209	*Acinetobacter*	*Acinetobacter radioresistens* (T); DSM 6976; X81666	1.000	0	0	0	0	0	0	12	0
OTU_1153	*Sulfurospirillum*	*Sulfurospirillum alkalitolerans (T), HTRB-L1, GQ863490*	0.743	5	19	15	41	89	0	131	0

The OTUs are shown classified at the genus level based on the RDP classifier (OTUs with scores > 70% confidence threshold were considered classified at the genus level). The closest type strain on RDP represents the closest type species at the RDP database and respective closeness score (a_b probability score). Note that OTU 209 was identified as species *Acinetobacter radioresistens.*

Of the 13 contaminating OTUs, one was only identified at the family level (meaning that it may belong to an unknown genus), three were only identified at the genus level, eight were closely related to a type strain (> 90% similarity), and one was identified at the species level with maximum confidence level from the software (Table 3). OTU 63 is closely related to human enterobacteria (*Shigella* spp.) and through this method, we were able to know that while this bacterium was present in the processing control, it was not detected in any of the environmental samples. Processing ice samples demands much more human contact than DNA extraction, agreeing with this result. OTU 98 is a close match for *Burkholderia stabilis*, notably a known contaminant of class I medical devices including gloves^44,45^. This OTU was also mostly present in the processing control. This result highlights how significant DNA contamination is when samples are handled by humans, even if properly equipped, and are then analyzed through sensitive DNA detection techniques. OTU 1153 and OTU 25 were the most representing contaminants in the environmental samples, with approximately 0.1% of average relative abundance among all environmental samples (see Table 2). OTU 25, closely related to *Acinetobacter guillouiae*, was highly abundant in the DNA-extraction control, in line with the genus-level taxonomic composition of this sample (Fig. 3). Some other OTUs present in Table 3 had low abundance (below detection limit) in the environmental samples while being detected in the DNA-extraction control. Previous analyses of contaminants from DNA extraction reagents showed the presence of extremophilic strains such as members from the radioresistant genus *Deinococcus*^41^. A species exclusive from the DNA-extraction control, OTU 209, was a 100% match to *Acinetobacter radioresistens*. This bacterium is highly tolerant to gamma-ray radiation^46^, thus difficult to eliminate with radiation-based sterilization. Overall, the DNA-extraction control rather than the processing control was a more disruptive source of contamination, suggesting that DNA extraction protocols for life-detection analysis will always profit from the use of background controls. A recent study on the contaminants from NASA Spacecraft Assembly Facility has shown the significance of the kitome in the field of planetary protection. It has also demonstrated that bacteria from human origin prevails in the kitome while bacteria known to survive extreme conditions were more likely contaminants from the clean room environment^43^. Our data unravel a not so evident pattern suggesting that some bacteria from human origin were more prevailing in the processing control (which would be more relatable to the environment of the laboratory) instead of as a member of the kitome, while the DNA extraction kit was a more likely source of the stress-resistant contaminating genus *Acinetobacter*. However, OTU 5, closely related with *Corynebacterium tuberculostearicum* type strain—part of the human skin microbiome, was found to prevail in the DNA-extraction control, likely being part of the kitome, agreeing with the previous NASA study^43^. These results highlight how relevant the disclosure and close monitoring of the communities present in controls, including the kitome, are for astrobiology analysis in planetary field analogs as well as future space missions.

## Applications to future space missions to the icy moons

Space is inhospitable for (most) known biological systems when considering the combination of vacuum conditions, extreme temperatures, microgravity, and, the most deleterious, unfiltered solar and galactic radiation^29^. The survival of terrestrial microbes during multi-year cruises to the outer solar system is unlikely, with best-case probability estimations of 6.98 × 10^−1^ for a mission to Europa^47^. However, for planetary protection requirements, these are still unacceptable values given that NASA Procedural Requirement (NPR) 8020.12D specify that the probability of a liquid extra-terrestrial water body must be less than 1 × 10^−4^^10^.While adding the probability of an unplanned impact strong enough to break the ice shell will decrease the overall chances of subglacial contamination^47^, forward contamination remains a concern for the exploration of icy worlds^10,48^. Future analysis on the equipment from missions directed to collect and return with samples from celestial bodies, such as Hayabusa2 and OSIRIS-REX will allow a closer assessment of probabilistic inferences^49–51^.

### Relevance to future missions to explore icy moons

The NASA Procedural Requirements (NPR) defines forward contamination in ocean worlds as “the introduction of a single viable terrestrial microorganism into a liquid–water environment”^10^. NASA Response to Planetary Protection Independent Review Board Recommendations (2019) advised the study of mechanisms of contamination individually for each of these worlds^52^, with more concern for Europa and Enceladus^10^. The environment of each icy moon (e.g., Europa, Enceladus, Ganymede, and Callisto) is very different from one another^4,53^ requiring different decontamination planning. Thus, there is an emerging need for well-established decontamination and background control-design protocols with years of proving data. The development of tools, such as the presented in this study, to monitor contamination in samples from icy terrestrial analogs will serve as a testbed for future search-for-life space missions while improving the fieldwork practices in glacial environments that face the same concerns.

Astrobiologists and microbial ecologists working with ice from planetary field analogs to icy moons should continue to share their contaminant identifications with the community. This has been established regarding Mars-related research^37,38,54,55^ and we recommend based on our findings that this practice should extend to icy moons as well. This way, the contaminating sources, and recurrent contaminants, if existing, of ice samples collected in the planetary field analogs to icy moons will be unraveled during the next years.

Drilling the thick ice shell of Europa and Enceladus surface, as conceived in Europa Lander, Enceladus Orbilander Mission, and JEM^6,7,56^, will require core recovery strategies analogous to those used in terrestrial studies, although substantially more complicated logistically. Decontamination techniques using UV-C and chemical disinfectants are suggested by Task Group on the Forward Contamination of Europa^10^ to sterilize hardware before the spacecraft launch to Europa. However, these agents are unsuitable to be used on the external layers of ice cores since they would change the putative extraterrestrial community’s abundance and diversity profiles^23,26^. Our results show how mechanical removal of external ice core layers is effective in reducing bioburden and not aggressive to the natural biota. We recommend that the design of ice corers for future missions should incorporate a mechanism to remove or degrade the external layer of the ice cores mechanically or through the application of heat.

The processing and downstream analysis for these missions is based on the ones used in planetary field analogs^57,58^. For instance, Enceladus Orbilander Mission is designed to use Nanopore sequencing innovation to detect life, a small device able to sequence long strands of DNA and RNA in real-time, identifying long polymeric strands of nucleic acids if present^7,59^. However, the use of amplicon techniques should still be considered in this field, for monitoring contamination^10^. Our prokaryotic monitoring success relied heavily on the power of PCR to detect contaminants in the controls. The increased sensitivity of this technique has been used to closely explore total communities, including the polar “rare biosphere”^60^, and has been applied to the analysis of ice samples in planetary field analogs to the icy moons^61–67^, as well as planetary protection investigations^18^. Thus, while long-strand sequencing will be necessary to characterize putative extraterrestrial DNA, amplicon sequence remains as preferential to monitor contaminations from Earth.

In a future analysis of planetary field analogs to prepare for space missions we recommend including replication in the design of the experiment to decrease PCR bias as well as allow for deeper assessment of the origin of contaminations. We also strongly reinforce the need for new research on functional metagenomics for characterizing the physiological potential of microbial contaminants, which will be beneficial for future decontamination techniques. While identifying the contaminant microorganisms may help the monitoring and trace contamination, knowing their survival strategies as a group, through the study of their functional roles, may prove useful for future improved broad-spectrum decontamination methods.

Returning samples have been described as a fundamental part of future landing missions in Europa and Enceladus^68,69^. In fact, Icy Moon Sample Return Mission is considered an “inspirator” by ESA to bring Europe’s space ambitions to the next level (ESA’s Intermediate Ministerial Meeting 2021). However, a planetary protection concern regarding return missions is that extraterrestrial life may contaminate Earth (backward contamination). Thus, future return missions fall into the V category (COSPAR planetary protection policy 2017)^8^, which is restricted in cases of Europa and Enceladus. The contamination controls for such missions need to avoid “false positive” indications in life-detection, the protocol for biohazards during cruise, landing, and after it is returned. “False positive” contamination could lead to unnecessarily increased rigor in the requirements for all later Europa or Enceladus missions and prevent the distribution of the sample from containment and their further study. A pre-condition for such missions is that a program of life detection and biohazard testing, or a proven sterilization process, shall be commenced for the controlled distribution of any portion of the sample. As opposed to our recommendations for the use of amplicon-approaches to monitor forward contamination, DNA-based techniques to detect backward contamination should not have a prime focus on the use of molecular markers since life may have evolved differently outside Earth. Thus, methods that allow high-resolution identification of nucleic acids chains without resorting to molecular markers are a preferred approach. Currently, Europe does not have a laboratory prepared to receive and analyze return extraterrestrial samples^70^. Thus, as recommended, new research based on the bulk use of standardized decontamination protocols in planetary field analogs to the icy moons is a key necessity to formulate curational procedures for such future laboratories to guarantee the maximum efficiency and safety of future extraterrestrial life-detection missions.

## Conclusions

Landing missions to Europa and Enceladus are at least two decades away. However, implementing clean sampling strategies for extraterrestrial life detection experiments, based on verifiable and standardized control methods used in planetary field analogs will take a long time. We recommend that the community does not only rely on the effects of the natural radiation environment of these moons for decontamination and incorporate the degradation of the external layers of ice cores contacting with man-made equipment in the mission’s design. We recommend microbial contamination monitoring on strategic checkpoints during these missions. Our results suggest that the most appropriate techniques to search for forward contaminants on extraterrestrial ice cores may not be the same considered appropriate to search for extraterrestrial life, and that the incorporation of both methodologies in future missions should be considered. Finally, we encourage the scientific community working on planetary field analogs to keep sharing their contaminants. Searching for a “core” contaminating microbiome of ice sampling and processing procedures from planetary field analogs will also take years of consistent research and public deposition of data, culminating in comparative studies. This represents an additional technological barrier to an already difficult mission. However, space missions require massive resources, and the best methods are needed to unambiguously convince the scientific community of the prospective discovery of the century—finding extraterrestrial life. Adding to contamination of an extraterrestrial world with terrestrial biota would have a severe impact with unquantifiable long-term costs, especially if this contamination is not immediately detected and monitored.

## Materials and methods

### Sampling site and collection of ice and water

Sampling was performed in the southeast of Hudson Bay, a coastal point 12 km away (latitude 55.39° N; longitude 77.61° W) from the Indigenous communities of Whapmagoostui–Kuujjuarapik in Nunavik, northern Quebec, Canada (Fig. 1A). First-year sea ice formations are characterized by a one-meter-thick shell on top of Hudson Bay’s water during winter, creating a highly dynamic freezing/melting cycle in the water–ice interface where nutrients and microbes accumulate^71^. A river system (Great Whale River) is relatively close to, discharges into and influences the surface bay waters with freshwater inputs, as commonly found in Arctic coastal marine systems^72^. The ice and the interface water (water just beneath the ice) of the sampled point in Hudson Bay are considered analogs to the water–ice interface environment found in Europa and Enceladus (water bodies perched in ice) owing to the low thickness of the first-year sea ice (1 m). Also, the salinity of the interface water was 1.05% by mass, being within the 0.5–4% interval of Enceladus^73,74^ and the interface water temperature was − 0.4 °C which is within the interval of the interface water temperature of both satellites (− 3.98 and 0 °C)^75,76^, either associated with perched liquid water reservoirs closer to the surface or even the internal ocean.

Samples were collected in late winter (Feb 27th, 2019) when ice, approximately 1-m thick, covered the bay. The snow cover (20 cm) was removed from the sampling site. An ice auger was used to drill a hole in the sea ice shell and the interface water was bottled right beneath the ice (1 L in triplicate) using a Kemmerer bottle with 2.2 L of volume (Fig. 1C). Ice cores were sampled in duplicate (50 cm apart and encompassing the interface water sampling site) using a Kovacs ice corer with 9 cm of diameter and 1 m of length. In total, five samples were collected: Ice 1, Ice 2, Water 1, Water 2, Water 3, all from the same site. The ice cores were handled with gloves and placed inside sterile bags. The lowest temperature registered at the weather station of Whapmagoostui-Kuujjuarapik during sampling days was − 28 °C, while the highest temperature reached was − 11 °C at noon, with an almost constant wind flow of 21 km/h. The salinity and temperature data were collected on-site using YSI EXO2 multiparameter probe (YSI Inc. Ohio, USA), which was placed in the interface water after extracting the ice cores.

### Design and construction of background controls

#### Construction of sterile artificial ice core–processing control

A sterile artificial ice core, denoted the “processing control, was constructed from 1 L of sterile Milli-Q water (Fig. 1D), which was double filtered through a Millipore system (18.2 MΩ cm, 25 °C) in the facilities of Centro de Química Estrutural (CQE), Instituto Superior Técnico (Portugal). The water was then doubled autoclaved at 121 °C for 30 min and irradiated for 45 min using the UV germicide lamp present inside a class II Microbiological Safety Cabinet (Faster BH-EN 2004), in the facilities of Institute for Bioengineering and Biosciences (iBB), Instituto Superior Técnico, Portugal. The Milli-Q water was carefully transported to Centre d’Études Nordiques (CEN), a research station located in Whapmagoostui-Kuujjuarapik, where it was solidified inside a sterile zipper bag placed in a cylinder shape container at − 60 °C before the collection of the environmental ice cores used as a comparison in this study. This sterile artificial ice core (processing control) was later processed in parallel with the environmental ice cores and interface water samples as described below. This background control allowed the monitoring of contamination from the instruments and procedure used to cut and bag the ice and filter the ice meltwater (see below).

#### Design of DNA-extraction control

A clean filter, deposited in a clean microtube was also subjected to the same total-community DNA (TC-DNA) extraction protocol (see below) as the environmental samples and the processing control to assess possible contamination in downstream analysis. Even considering that only material with adequate sterility pre-treatment for molecular biology and specific for DNA analysis was used (DNA-, DNase- and PCR-inhibitors free plastics and reagents as well as filtered tips), it is important to evaluate possible contamination from the extraction materials and reagents used as well as the general environment of the laboratory^40^. This blank DNA extraction is here called the “DNA extraction control” (Fig. 1D).

### Sample decontamination procedure and processing

Sample decontamination and processing took place in the laboratory facilities available at Centre d’Études Nordiques (CEN), using sterile equipment (autoclaved, irradiated, and/or washed with ethanol). The external layer (5 mm) of the ice cores was cut using an ice saw (pre-treated with ethanol) and then rinsed with autoclaved, irradiated, and doubled-filtered MilliQ water to reduce handling, air, and snow contamination as well as contamination from the sampling materials in the field. The remaining inner core was rinsed with sterile MilliQ water and left melting at room temperature in sterile bags (Fig. 1B). Ice meltwater from environmental ice samples (1000 mL), ice meltwater from the processing control (500 mL) and interface water samples (500 mL) were filtered through 0.22 µm nitrocellulose filters (Millipore®, Sigma-Aldrich) using a vacuum pump (Fig. 1B). Before downstream analysis (DNA extraction, amplification, and sequencing), the filters remained frozen and were stored at − 80 °C. Unfiltered ice meltwater and interface water were stored in sterile bottles and maintained at 4 °C until cultivation.

### Total community DNA extraction and quantification

TC-DNA extractions were performed on the filters of both the environmental samples (Ice1, Ice2, Water1, Water2, Water3) the background controls (processing control and DNA-extraction control) at iBB in Portugal. The TC-DNA extraction was carried out as described previously^28,77^. The bench was previously cleaned with both ethanol (70%) and HCl (0.1 M). Filters were cut into small pieces to ensure full submersion and improve the contact between the cells and lysis buffer and enhance the probability of extracting low-abundance DNA (due to the nature of the ice samples). The Ultraclean Soil DNA Kit (MoBio Laboratories, Carlsbad, CA, USA) was used for DNA extraction, following the manufacturer’s protocol, for offering vigorous membrane lysis, adequate for ice samples that typically house multiple sporulated microorganisms. Agarose gel electrophoresis procedures were used to examine the integrity of TC-DNA, not showing any DNA bands under UV radiation for either the processing control or DNA-extraction control. TC-DNA concentrations estimations were quantified using the Qubit 4 Fluorometer with the high-sensitivity dsDNA assay kit (Invitrogen, CA, USA) with a detection limit of 10 pg/µL.

### DNA sequencing and processing

#### High-throughput sequencing of 16S rRNA genes and processing of sequencing data

The TC-DNA extracts were subjected to 16S rRNA gene-based high-throughput sequencing on an Illumina MiSeq platform at MR DNA following the company’s protocol (www.mrdnalab.com, Shallowater, TX, USA) and using prokaryotic universal primers (for both bacterial and archaeal identification) 515F (5′ -GTG YCA GCM GCC GCG GTAA-3′) and 806RB (5′-GGA CTA CNV GGG TWT CTA AT-3′)^78,79^. During sequencing, an average of around 300 bp (base-pairs) sequences were generated for all samples as described in Coelho et al.^28^. An average quantity of 23,000, 21,000, and 9000 paired-end sequences were generated per environmental interface water sample, background control sample, and environmental ice sample, respectively.

The 16S rRNA gene amplicon libraries generated in this study were processed within the framework of a previous microbial ecology study on the Hudson Bay coastal ice^28^ and deposited in the open-source online metagenomic repositorium European Nucleotide Archive (ENA) under the study accession number [PRJEB44116]. Briefly, raw sequences with less than 150 bp and with ambiguous base calls as well as chimeric sequences and singletons, were removed as described earlier^80^. OTUs were generated and defined by clustering at 3% divergence (97% similarity) with the UCLUST algorithm. A taxonomic classification of OTUs using the SINA sequence alignment tool (v1.2.11) of the SILVA database (https://www.arb-silva.de/aligner/) was performed. The OTUs displaying less than 70% identity were considered as unclassified at the domain level and removed from the data, eukaryotes (3 OTUs), chloroplasts (147 OTUs), mitochondria (9 OTUs) reaching a total of 159 were also removed. The final analytical dataset comprised 2331 prokaryotic OTUs and 134,230 sequence reads.

#### In silico decontamination

In silico decontamination procedures^27,40^, consisted of the removal of all OTUs detected (through sequencing and data processing—see below) in the previously described background controls from the environmental samples. Different in silico decontamination techniques may be applied such as the exclusion of OTUs whose relative abundance in background controls is above a given threshold; exclusion of OTUs with high relative abundance; exclusion of OTUs with higher relative abundance in background controls than in the environmental samples; or, more conservatively, remove all OTUs present in the background controls from the results of the environmental samples^40^. Due to the application of this study in astrobiology and NASA recommendations on planetary protection, we assumed the most conservative approach of eliminating all OTUs also present in the background controls.

### Analysis of taxonomic composition and beta-diversity

Stacked bar charts displaying the taxonomic composition were generated based on the relative abundance of prokaryotic taxa from phylum to genus ranks. Low abundance taxa (e.g., taxa below 0.5% of relative abundance) were merged into a single category called “Others” to improve the readability of the environmental samples which, as expected, were substantially more abundant than the background control samples. Venn diagrams were generated using the VennDiagram package in R to determine the number of OTUs specific to and shared by the environmental samples and the background control samples. The plots were created using R (v. 4.1.0) packages *phyloseq*, *plyr,* and *ggplot2*. Non-rarefied data was used to prevent loss or ill-representation of low abundance OTUs, an important parameter for this study^81^. The taxonomic annotation of the OTUs present in the background controls (processing control and DNA-extraction control) were further accessed using the Ribosomal Database Project (RDP)^82^ applying the tools “Classifier” for genus assignment and the tool “Sequence Match” to search for the closest type strain. Type strains were chosen because they are less likely to be poorly identified, giving us accurate information regarding the closest species found in the database. Taxonomic assignments by the RDP “Classifier” below the 70% confidence threshold were considered unclassified.

### Sample cultivation in R2A medium and CFU counts

Culturing was performed at iBB (Portugal) after transporting (48 h) the environmental interface water and ice meltwater as well as the processing control samples, inside sterile bottles in a dark cooler at the average temperature of 4 °C. Interface water samples were serially diluted and cultured by spread-plating on 1:10 R2A agar medium, while 100 µL of both environmental ice meltwater and the processing control samples were directly spread on 1:10 R2A agar medium^83^. Water samples were cultured with R2A dissolved in sterile artificial seawater (ASW) and ice samples (environmental ice meltwater and processing control), were cultured with R2A dissolved in sterile MilliQ water. The two culture settings were designed due to: (1) the estuarine nature of the sampled environment, (2) the variable saline concentrations of ice, and (3) to widen putative possibilities of obtaining colonies from the processing control (a low-biomass sample) while still being able to compare the results with the environmental ice sampled. Spread plating was performed in triplicates (interface water) and duplicates (ice meltwater and processing control) per dilution. Plates were then incubated at 15 °C, and CFUs were counted every 5 days for 30 days. As a procedure control, clean plates were open during the culturing procedures, kept for 30 days, and no growth was registered.

## Supplementary Information

Supplementary Information.

## Data Availability

The dataset analyzed in this study is available in the European Nucleotide Archive (ENA) under the study accession number [PRJEB44116] with the sample accession numbers [SAMEA8547042–SAMEA8547046; SAMEA85459-SAMEA85460] and the run accession numbers [ERR5696667-ERR5696671; ERR5696684-ERR5696685].
