# Supplementary material for: Contamination analysis of Arctic ice samples as planetary field analogs and implications for future life-detection missions to Europa and Enceladus
Source: Sci Rep. 2022 Jul 27;12:12379. doi: 10.1038/s41598-022-16370-5 (PMC9329357; doi:10.1038/s41598-022-16370-5)
Supplement: Supplementary file 1 — Supplementary Information. [file 41598_2022_16370_MOESM1_ESM.docx]

**Contamination analysis of Arctic ice samples as planetary field analogs and implications for future life-detection missions to Europa and Enceladus**

Lígia F. Coelho^1,2,3^, Marie-Amélie Blais^4,5^, Alex Matveev^4,6^, Tina Keller-Costa^2,3^, Warwick F. Vincent^4,5^, Rodrigo Costa^2,3,7^, Zita Martins^1*^, João Canário^1*^

**Supplementary information**

**
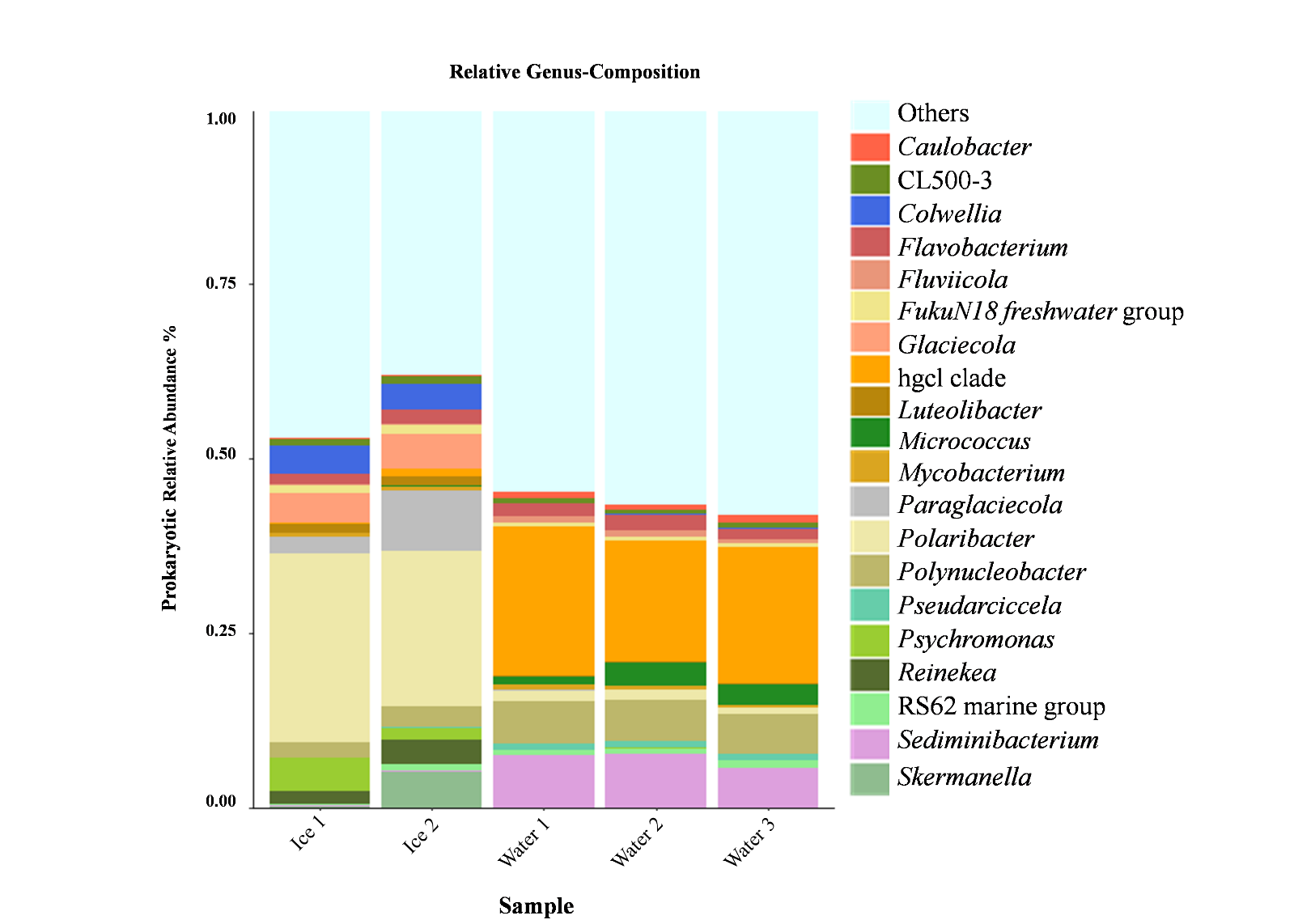
**

**Figure S1.** Taxonomic composition of prokaryotic communities in environmental samples (ice and interface water, including replicates) after *in silico* decontamination process where all OTUs present in the controls were removed. Results based on the relative abundance of OTUs of the non-rarefied dataset. For improved readability, taxa below 0.5% of relative abundance were combined under the category "Others". Note that groups such as *Acinetobacter* and *Sulfurospirillum* are absent from the taxonomic profiles of these samples after removing contaminating OTUs.

**Table S1.** **Evolution of techniques used for contamination control during the sampling, processing, or biological analysis of ice cores from the last 23 years. Artificial sterile ice cores, also known as “sham” or “mock” ice cores, are made in the laboratory, and used as controls. N.A. means “non-available”. Culture-dependent analysis refers to analysis performed on cultured microorganisms (e.g., colonies or liquid cultures). Culture-independent analyses include molecular biology studies, microscopy, and flow cytometry. This table was based on Table 1 from Christner et al., (2005)^20^ representing a prolongation to more recent days.**

| **Origin** | **Type of ice** | **Control** | **Replicates** | **Decontamination procedures** | **Analysis performed** | **Ref.** |
| --- | --- | --- | --- | --- | --- | --- |
| Ellesmere Island  (Canadian Arctic) | Glacier ice | N.A. | N.A | Sterile materials; Heat; Ethanol (95%) used on equipment/material the filtration assembly were sterilized between samples | Culture-dependent analysis | **33** |
| Vostok 5G ice core (Antarctica) | Lake accretion ice | N.A. | One ice core, ten ice sections | Sterile materials; Heat | Culture-dependent analysis | **34** |
| Deep ice core – GISP2 (Greenland) | Glacier ice | Artificial sterile water ice core | N.A. | Sterile materials; Superficial layer removal (2-3mm); Ethanol (95%); Superficial layer removal (1 cm); Heat | Culture-independent and culture-dependent analysis | **35, 61** |
| Tallasksenvarden Nunatak (Antarctica) | Glacier ice | N.A. | N.A. | Sterile materials; Superficial layer removal | Culture-dependent analysis | **36** |
| Vostok 5G ice core (Antarctica) | Lake accretion ice | Sterile water;  Lake ice core water sterilized | One ice core, four ice sections | Sterile materials; Sodium hypochlorite solution at 4ºC; Sterile water | Culture-independent analysis | **62, 64** |
| Ellesmere Island  (Canadian Arctic) | Sea ice | N.A. | Two ice cores | Sterile materials, the filtration assembly was sterilized between samples | Culture-independent analysis | **65** |
| Muztagh Glacier  (Tibetan Plateau, China) | Glacier ice | N.A. | N.A. | Sterile materials; Superficial layer removal; Ethanol (95%); Sterile water | Culture-dependent analysis | **37** |
| Cornwallis Island  (Canadian Arctic) | Sea ice | N.A. | Eight ice cores per location | Sterile materials | Culture-independent analysis | **63** |
| Yuzhufen Glacier  (Tibetan Plateau) | Glacier ice | N.A. | One ice core, 4 sections, 1253 sub-sections | Sterile materials; Superficial layer removal; Sterile water; Ethanol (75%) | Culture-dependent analysis | **38** |
| Matanuska Glacier (Alaska) | Glacier ice | N.A. | N.A. | Sterile materials; Superficial layer removal (5 mm); Sterile water; Ethanol (95%) | Culture-independent analysis | **66** |
| Changme Khang and Changme Khangpu glaciers (India) | Glacier ice | N.A. | One ice core per glacier | Sterile materials; Superficial layer removal; Sterile water; Ethanol (95%) | Culture-dependent analysis | **39** |
| Scarisoara Ice Cave (Romania) | Perennial cave ice | N.A. | One ice core, five ice sections | Sterile materials | Culture-independent analysis | **67** |
| Guliya ice cap  (Tibet) | Glacier | Artificial sterile water ice core; Air control, DNA extraction control; Standard 16S rRNA gene amplicon sequencing of controls | One ice core, five sections | Sterile materials; Superficial layer removal; Ethanol 95% and new layer removed; Sterile water | Culture-independent analysis | **27** |
| Hudson Bay  (Canadian subarctic) | Sea ice | Artificial sterile water ice core; DNA extraction control; Standard 16S rRNA gene amplicon sequencing of controls | Two ice cores | Sterile materials; Superficial layer removal; Ethanol 95%; Sterile water | Culture-dependent and culture-independent analysis | **28** |
